# Stable human cartilage progenitor cell line stimulates healing of meniscal tears and attenuates post-traumatic osteoarthritis

**DOI:** 10.3389/fbioe.2022.970235

**Published:** 2022-10-12

**Authors:** Salomi Desai, Mark Dooner, Jake Newberry, John Twomey-Kozak, Janine Molino, Jay Trivedi, Jay M. Patel, Brett D. Owens, Chathuraka T. Jayasuriya

**Affiliations:** ^1^ Department of Orthopaedics, Warren Alpert Medical School of Brown University and the Rhode Island Hospital, Providence, RI, United States; ^2^ Department of Medicine, Division of Hematology Oncology, Rhode Island Hospital, Providence, RI, United States; ^3^ Department of Orthopaedics, Emory University, Atlanta, GA, United States

**Keywords:** meniscus repair, cartilage progenitor cell, stem cell, progenitor cell, cartilage, PTOA, regenerative medicine, rodent model

## Abstract

Meniscal tearing in the knee increases the risk of post-traumatic osteoarthritis (OA) in patients. The therapeutic application of tissue-specific mesenchymal progenitor cells is currently being investigated as an emerging biologic strategy to help improve healing of musculoskeletal tissues like meniscal fibrocartilage and articular hyaline cartilage. However, many of these approaches involve isolating cells from healthy tissues, and the low yield of rare progenitor populations (< 1% of total cells residing in tissues) can make finding a readily available cell source for therapeutic use a significant logistical challenge. In the present study, we investigated the therapeutic efficacy of using expanded cartilage-derived and bone marrow-derived progenitor cell lines, which were stabilized using retroviral SV40, for repair of meniscus injury in a rodent model. Our findings indicate that these cell lines express the same cell surface marker phenotype of primary cells (CD54+, CD90+, CD105+, CD166+), and that they exhibit improved proliferative capacity that is suitable for extensive expansion. Skeletally mature male athymic rats treated with 3.2 million cartilage-derived progenitor cell line exhibited approximately 79% greater meniscal tear reintegration/healing, compared to injured animals that left untreated, and 76% greater compared to animals treated with the same number of marrow-derived stromal cells. Histological analysis of articular surfaces also showed that cartilage-derived progenitor cell line treated animals exhibited reduced post-traumatic OA associated articular cartilage degeneration. Stable cell line treatment did not cause tumor formation or off-target engraftment in animals. Taken together, we present a proof-of-concept study demonstrating, for the first time, that intra-articular injection of a stable human cartilage-derived progenitor cell line stimulates meniscus tear healing and provide chondroprotection in an animal model. These outcomes suggest that the use of stable cell lines may help overcome cell source limitations for cell-based medicine.

## Introduction

The menisci are crescent shaped fibrocartilaginous tissues (Makris et al., 2011) that help to distribute axial load and provide knee joint stability (Fox et al., 2012). Meniscus injuries account for up to 20% of total knee injuries in young athletes, for whom the most sophisticated epidemiological data exist (Cameron et al., 2017; Kennedy et al., 2020). Specifically, meniscus tearing is one of the most common knee injuries occurring in 6—8% of active young adults annually in the U.S. (Baker et al., 1985; Jones et al., 2012) and it results in increased incidence rate of osteoarthritis (OA) (Englund et al., 2009). Tears compromise the ability of the meniscus to distribute loads in the joint, increasing cartilage-cartilage stresses, and starting a degenerative cascade. These injuries are treated clinically by arthroscopic surgical resection or suture repair, based on the nature and extent of tearing. However, due to the relatively hypovascular and hypocellular nature of meniscal fibrocartilage, its intrinsic healing capacity is limited, which is reflected in a high reoperation rate after meniscal repair of 16.5%–20.7% (Paxton et al., 2011). For these reasons, better stimulation of meniscal fibrocartilage healing could reduce reoperation and post-traumatic OA (PTOA) progression.

Mesenchymal progenitor cells derived from healthy articular cartilage, herein referred as CPCs, are a small subset of cells that are highly proliferative, chondrogenic, and migratory (Dowthwaite et al., 2004; Seol et al., 2014; Jayasuriya and Chen, 2015; Jayasuriya et al., 2016), making them a potentially useful resource for biologic cell therapy and cartilaginous tissue engineering applications (McCarthy et al., 2012; Seol et al., 2014; Bilgen et al., 2018; Park et al., 2020). Further, they are resistant to cellular hypertrophy and terminal differentiation (McCarthy et al., 2012; Twomey-Kozak et al., 2020), unlike mesenchymal progenitor/stem cells from bone marrow and osteoarthritic (diseased) cartilage tissue (Jayasuriya et al., 2018; Hu et al., 2019; Liu et al., 2020). We have recently demonstrated the efficacy of using stable CPC lines, either as such or in a collagen coated hydroxypropyl cellulose scaffold, to stimulate meniscus injury repair in rat and human *ex-vivo* organ culture model systems, respectively (Jayasuriya et al., 2019; Newberry et al., 2020).

In this study, we sought to validate our previous *ex-vivo* findings using a live animal model. To this end, our objective was to test the efficacy of intraarticularly injecting CPCs as a biologic cell therapy in order to accelerate healing of a meniscal tear injury in live rats. In particular, we hypothesized that CPC injections would accelerate meniscal healing and reduce the progression of PTOA. We opted to use one of the four previously generated and characterized stable CPC lines (CPCL3) for this purpose, considering the low natural abundance of primary CPCs in healthy articular cartilage (Jayasuriya et al., 2019). Our motivation was to report on the therapeutic efficacy of using this abundant and expandable CPC source for cartilaginous musculoskeletal tissue repair.

## Materials and methods

### Isolation and generation of human progenitor cell lines

The stable CPC line (CPCL3) used in this study was generated previously (Jayasuriya et al., 2019). A stable BM-MSC line (BM-MSCL) was generated in the same way by infecting with pRetro-E2 SV40 (Applied Biological Materials, Inc., Richmond, BC, Canada) according to the manufacturer. Primary CPCs (P-CPCs) were isolated from the non-arthritic articular cartilage of three different patients: 15-year, 18-year and 42-year-old, undergoing arthroscopy, with an approval from the IRB of Rhode Island Hospital (committee number 0070–17). Cartilage was diced followed by enzymatic tissue digestion using Pronase (Roche, Indianapolis, IN; 2.0 mg/ml in HBSS) and Collagenase type IA (Sigma-Aldrich, St. Louis, MO; 1.0 mg/ml in HBSS), similar to previously described method (Jayasuriya et al., 2019). P-CPCs were enriched by fibronectin adhesion (Williams et al., 2010; Jayasuriya et al., 2019) and adherent cells were cultured in Dulbecco’s Modified Eagle’s Medium supplemented with 10% Fetal Bovine Serum (FBS), 1% Pen Strep, 100 mM HEPES, 2 mM l‐glutamine, 0.1 mM ascorbic acid, 0.1 mM sodium pyruvate, and 2.7 μM l‐glucose (DMEM++) media and used for experiments as P-CPCs. Human Bone Marrow Mesenchymal Stem Cells (BM-MSCs) were purchased from American Type Culture Collection (ATCC) (PCS-500–012). Both BM-MSCL and CPCL3 were cultured and expanded in DMEM++ media for experiments conducted throughout this study.

### Cell surface marker analysis

Cell surface marker analysis was performed to compare primary cells to stable cell lines. Cell suspensions were stained with antibody pre-conjugated with dye. CD90-FITC/PE (catalog # 130-114-901/130-114-902), CD54-PE (catalog # 130-120-780), CD49e-PE (catalog # 130-10-590), CD166-APC (catalog # 130-104-164), CD105-APC (catalog # 130-099-125), and IgG isotype-FITC/PE/APC controls (catalog # 130-113-761/130-113-762/130-113-758) from Miltenyi Biotec Inc., Auburn, CA. were used according to manufacturer’s protocol. Cells were washed in 5 ml of 1X PBS, spun down in a centrifuge at 300 g and resuspended in 100 μl of buffer (1X PBS, 0.5% Bovine Serum Albumin). Pre-conjugated antibody (10 μl) was added to the cell buffer and incubated in the dark at 4°C for 10 min. Flow cytometry was performed using an LSRII flow cytometer (BD Biosciences, Franklin Lakes NJ), and data were analyzed using FlowJo, Version10.7.2, (BD Biosciences, Franklin Lakes, NJ).

### RNA expression analysis

Messenger RNA (mRNA) expression was quantified using real-time quantitative polymerase chain reaction (RT-qPCR). Total mRNA was isolated from cells *via* MagMAX™-96 for Microarrays Total RNA Isolation Kit (Thermo Fisher Scientific, Waltham, MA), according to manufacturer’s protocol. mRNA was reverse transcribed into cDNA using iScript cDNA Synthesis Kit (Bio-Rad, Hercules, CA), according to manufacturer’s protocol. RNA levels were calculated using the delta delta Ct (ΔΔCt) method and normalized to beta-actin, housekeeping gene. X = 2 ^-∆∆Ct^, in which ∆∆Ct = (Ct _Exp target gene_–Ct _Exp housekeeping gene_)—(Ct _Ctl target gene_–Ct _Ctl housekeeping gene_) and X = Relative transcript; Ct _Exp_ = Ct of experimental group, Ct _Ctl_ = Ct of control group. Data obtained from the three patient derived primary CPCs were pooled in order to compare their mRNA profile to that of CPCL3. Primer sequences used to test each gene are listed in Table 1.

**TABLE 1:** List of forward and reverse primers, in 5’ to 3’ orientation, used for Real-Time Quantitative PCR. NCBI accession identification numbers of target sequences are included for each primer pair.

Gene	Forward seq.	Reverse seq.	Accession
SOX9	GGA​CCA​GTA​CCC​GCA​CTT​GCA	GTT​CTT​CAC​CGA​CTT​CCT​CCG​CCG	NM_000346.3
COL1A1	CAG​GAG​GCA​CGC​GGA​GTG​TG	GGC​AGG​GCT​CGG​GTT​TCC​AC	NM_000088.3
COL2A1	CTC​CCA​GAA​CAT​CAC​CTA​CCA​CT	CGT​GAA​CCT​GCT​ATT​GCC​CT	NM_001844.4
Beta-Actin	GGA​CCT​GAC​TGA​CTA​CCT​CAT	CGT​AGC​ACA​GCT​TCT​CCT​TAA​T	NM_001101.4
MMP2	AGG​AGG​AGA​AGG​CTG​TGT​TC	TGG​GGA​AGC​CAG​GAT​CCA​TT	NM_004530.6
MMP13	ATG​CGG​GGT​TCC​TGA​TGT​GG	GGC​CCA​GGA​GGA​AAA​GCA​TG	NM_002427.4
ADAMTS-4	CCC​CAG​ACC​CCG​AAG​AGC​CA	CCC​GCT​GCC​AGG​CAC​AGA​AG	NM_005099.6
ADAMTS-5	GGC​CGT​GGT​GAA​GGT​GGT​GG	GCT​GCG​TGG​AGG​CCA​TCG​TC	NM_007038.5
Mitochondrial Cytochrome B	TAG​CAA​TAA​TCC​CCA​TCC​TCC​ATA​TAT	ACT​TGT​CCA​ATG​ATG​GTA​AAA​GG	NC_012920.1

### Cell proliferation analysis

Cell proliferation assay was used to measure rates of proliferation between primary cells and cell lines. Cells were cultured for 48 h, and cell proliferation was measured using Click-iT™ Plus EdU Alexa Fluor™ 488 Flow Cytometry Assay Kit (Thermo Fisher Scientific, Waltham, MA), according to manufacturer’s protocol. Cells (5.0 × 10^5^) were stained with EdU for 2 h, fixed and then analyzed using Flow Cytometry. Data was analyzed using FlowJo.

### Rodent meniscus injury model and treatment

All procedures used in this study were approved by the Institutional Animal Care and Use Committee (IACUC) of Rhode Island Hospital (committee number 0190–16). Skeletally mature male athymic RNU rats (12–15 weeks of age) were allowed to acclimate for 1 week prior to the surgery. Animals were anesthetized with isoflurane (3–5%) and the right knee was shaved and prepped thrice with surgical scrub followed by 70% alcohol. Pre-operatively, animals received cefazolin (20mg/kg) *via* intra-muscular injection as a prophylactic antibiotic and sustained release buprenorphine (1.2 mg/kg body weight) *via* subcutaneous injection to provide 72 h of analgesia. A medial side parapatellar arthrotomy was performed (by blunt dissection to minimize bleeding) on the right knee joints until the outer rim of the medial meniscus was exposed. Starting from the back rim of the medial meniscus, a full thickness 1.0 mm tear spanning from the red-red zone to the red-white zone was made using a micro scissor. The meniscus was left partially torn, and we ensured that it was not completely separated into two independent pieces. After the meniscal injury was induced, the synovial capsule was closed using 4–0 sutures, followed by muscle closure, and finally skin closure. VetBond skin glue was applied topically on the skin as an additional measure to close the wound site. Post-operatively, rats were housed two per cage and were permitted to bear weight as tolerated. Intra-articular injections of the CPCL3 or BM-MSCL treatments (1.6 × 10^6^ of cells in 50 µL sterile PBS), were administered twice, at Week 1 and Week 4 post-surgery, through the patellar tendon into the joint space. We selected to inject 50 µL because studies have shown that up to 100 µL can be injected into the knee joints of rats (Yang et al., 2015; Fan et al., 2018). Administered cells were fluorescently labelled with Vibrant CM-Dil Cell-Labeling (Cat: V22888, Thermo Fisher Scientific, Waltham, MA) solution according to the manufacturer’s instructions. The control group was administered with only the vehicle (50 µL of sterile PBS). Animals were euthanized 7-week following surgery for the postmortem analysis of relevant tissues.

### Human DNA analysis

Athymic rats were euthanized 7 weeks after surgery and their organs (kidney, lungs, liver, spleen, and brain) were harvested and frozen immediately. These tissues were weighed and homogenized by Proteinase K digestion using DNeasy Blood and Tissue kit (Qiagen, Hilden, Germany), and DNA was isolated using the provided mini spin column according to the manufacturer’s protocol. DNA was then quantified spectrophotometrically and used as template for PCR amplification of the human mitochondrial cytochrome B gene. Primer sequences for the gene product were obtained from Matsuda et al., 2005 (Matsuda et al., 2005). Amplified product was subjected to agarose gel electrophoresis and bands were observed under the UVP Bio-Doc Transilluminator.

### Histology analysis

Seven weeks following surgery, athymic rats were euthanized, and their medial menisci, femoral condyles and tibial plateaus were harvested, and fixed in 10% Neutral Buffered Formalin followed by decalcification and processing. Samples were sectioned at 4 µm in transverse plane for histological assessment of the meniscus and in parasagittal plane for articular cartilage. Menisci were stained with Saf-O/Fast Green and adjacent sister sections (collected approximately every 150 µm) were stained with DAPI. Femoral condyle and Tibial Plateau were stained with Saf-O/Fast Green. Scoring of the femoral condyle and tibial plateau was conducted using Modified Mankin scoring. Histological scoring was performed independently by two different graders that were blinded to the animal identifier and their respective surgical treatment group. Their mean scores were used for analysis.

### Statistical analysis

Statistical analysis was conducted using SAS version 9.4 (SAS Institute Inc., Cary, NC). Generalized linear models were used to compare all outcome measures between experimental conditions. Raw cycles-to-threshold of the synovial RT-qPCR were analyzed using a generalized linear model (GLM) with a Gaussian distribution and log link function, normalizing to the housekeeper gene within the model. The standard 2-to-the-negative-delta-delta formula was applied to the means and confidence intervals of relevant effects for presentation as normalized fold differences. GLM with a binomial distribution and logit link function was used to examine differences in Mankin score between experimental conditions and broken menisci while GLM with a Gaussian distribution and log link function was used to examine differences in the quantified size of defect area between experimental conditions. In the defect area analysis for the quantification of the size of the defect area was operationalized in two ways. In the first operationalization, animals in the 0-week control group, which were used to determine the reproducibility of the tear size, and animals with broken tears were excluded from the analysis since 7-week follow-up period was not collected for this set. In the second operationalization, 0-week control group was again excluded for the same reason and additionally, the missing value for the animals with broken tears was replaced with a variable that was 20% more than the maximum open area observed in the study. In all models, pairwise comparisons between experimental conditions were conducted within the GLM *via* orthogonal contrasts. The Holm test was used to protect against multiple comparisons and maintain a two-tailed familywise alpha of 0.05. Classical sandwich estimation was used to protect against possible model misspecification. Error bars in figures represent +1 standard deviation (SD) of the mean.

## Results

### Stable human mesenchymal progenitor cell lines maintain same cell surface marker profile as primary cells

**FIGURE 1:**
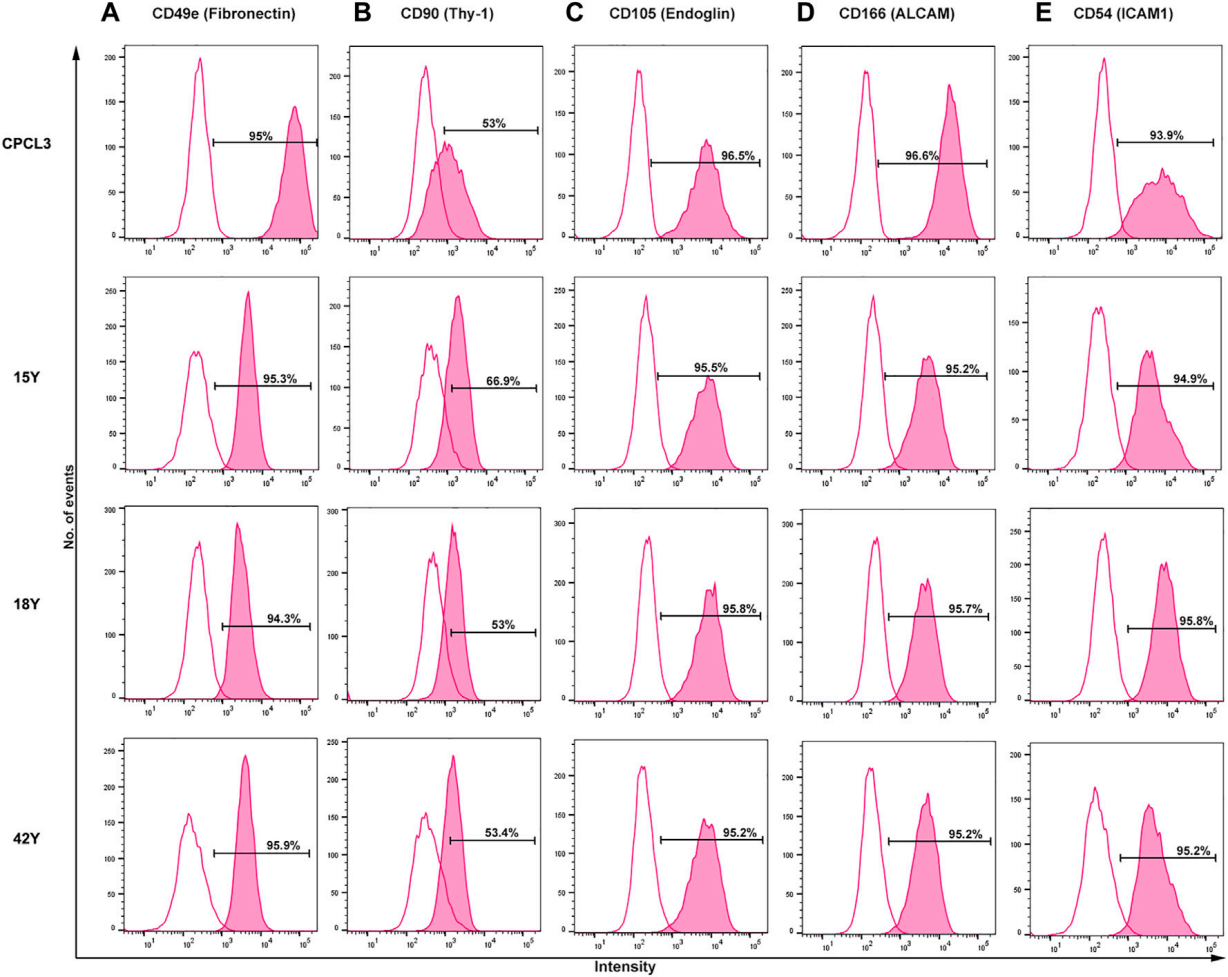
Side-by-side comparison of the cell surface marker profiles of the stable CPC line (CPCL3) and primary CPCs. Cell surface marker expression rates of **(A)** Fibronectin, **(B)** Thy-1, **(C)** Endoglin, **(D)** ALCAM, and **(E)** ICAM1 in CPCL3 and three primary CPC cell sources. Primary CPCs were independently collected and analyzed from three different individuals: 15-, 18-, and 42-years-old patients with no signs of degenerative joint diseases, such as osteoarthritis or rheumatoid arthritis. The empty peaks represent isotypic controls, for reference. Percent values indicated in the graphs represent positive staining rate, above isotype control, for each individual CD marker.

In order to test the *in vivo* efficacy of treating meniscus tears with CPCs, we utilized the human cartilage progenitor cell line 3 (CPCL3), which was first reported for its ability to stimulate meniscal tear reunion, in explant culture (Jayasuriya et al., 2019). In the present study, we generated a human BM-MSC cell line (BM-MSCL) to which the *in vivo* repair efficacy of CPCL3 can be compared. We decided to use BM-MSCs as our control because they are among the most commonly studied progenitor populations that are used in pre-clinical investigations of cell-based musculoskeletal tissue repair. BM-MSCL was created using retroviral gene transfer of Large T antigen, which is the same procedure used to generate CPCL3. Flow cytometry results indicating the cell surface marker profiles of CPCL3 showed no notable differences from primary cells, suggesting that it exhibits phenotypic stability during monolayer culture expansion (Figure 1).

First, we confirmed the presence of cell surface fibronectin receptor (CD49e), which is a selection criterion for progenitor cells in cartilage (Williams et al., 2010). CPCL3 and primary CPCs derived from three females (15, 18, and 42 years old) showed ≥ 94% positivity for CD49e cell surface expression. Next, we analyzed mesenchymal progenitor cell associated markers Thy-1 (CD90), Endoglin (CD105) and ALCAM (CD166) as well as chondrocyte associated marker ICAM1 (CD54). CD90 showed positive expression in >50% of the cells constituting the stable CPC line, as previously reported (Jayasuriya et al., 2019; Twomey-Kozak et al., 2020), and in the primary CPCs isolated from all three patients. CD105 and CD166 were expressed in ≥ 95% of the stable CPC line and all tested primary CPCs. Chondrocyte associated marker ICAM1 (CD54) (Kienzle and von Kempis, 1998; Lee et al., 2009) was also highly expressed in all CPCs tested (≥93%).

Cell surface marker profiling of BM-MSCL was also performed and compared to that of primary BM-MSCs (Supplementary Figure S1). Results showed that the stable line exhibited no significant deviations in all tested markers (CD49e, CD54, CD90, CD105, and CD166) suggesting that BM-MSCL is phenotypically stable and does not deviate from its primary counterpart, with regards to its cell surface marker profile.

### Stable CPCs exhibit lower collagen expression but higher *SOX9* expression and elevated proliferation capacity than primary CPCs

**FIGURE 2:**
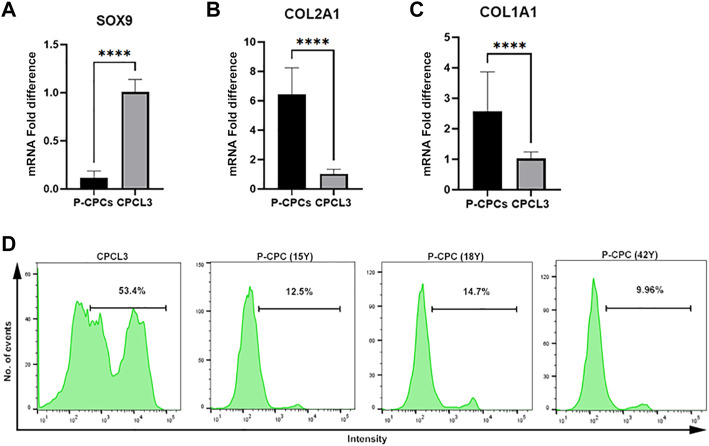
Comparison of chondrogenic marker expression and cell proliferation capacities of CPCL3 and primary CPCs. Fold differences in the mRNA expression of **(A)** *SOX9*, **(B)** type 2 collagen (*COL2A1*), and **(C)** type 1 collagen (*COL1A1*) between primary CPCs and the stable CPC line, CPCL3. N ≥ 4 per group. ****, *p* ≤ 0.0001. **(D)** Cell proliferation assay results showing the proportion of proliferating cells in CPCL3 cultures and primary CPC cultures obtained from three different patients.

Gene expression profiling of master chondrogenesis regulator *SOX9* and collagens, which are central to the fibrocartilage extracellular matrix network composition, was performed *via* RT-qPCR in the stable cell lines and primary cells. The stable CPC cell line exhibited higher basal *SOX9* expression compared to that of primary CPCs (Figure 2A), and lower expression of type II and I collagen (*COL2A1, COL1A1*) (Figures 2B,C). Primary CPCs expressed approximately a 6-fold increase in *COL2A1* and a 2.5-fold increase in *COL1A1*, relative to CPCL3. BM-MSCL was also compared to primary BM-MSCs with regards to the expression of these key mediators of cartilage and fibrocartilage ECM synthesis. We found that primary BM-MSCs exhibited a 4-fold increase in *SOX9* over BM-MSCL (Supplementary Figure S2A). Primary BM-MSCs expressed approximately an 8-fold increase in *COL2A1* (Supplementary Figure S2B) and a 2.5-fold increase in *COL1A1*, relative to BM-MSCL (Supplementary Figure S2C).

The cell proliferation capacities of CPCL3 and BM-MSCL was compared to that of their primary cell counterparts, respectively. Primary CPCs and BM-MSCs show a reduced proliferation capacity following *in vitro* monolayer culture expansion, in comparison to the stable cell lines and measured by EdU cell proliferation analysis using flow cytometer (Figure 2D, Supplementary Figure S2D). Among analyzed CPCL3 cultures, the proportion of cells that stained positive for EdU was 53.4%, whereas primary CPCs ranged from 9.96—14.7% (Figure 2D). BM-MSCL cultures contained 37.5% of cells that stained positive for EdU, while only 2.6% of primary BM-MSCs stained positive (Supplementary Figure S2D).

### Intra-articular injection of stable human cartilage-derived progenitor cells accelerate healing of a meniscal injury in athymic rats

**FIGURE 3:**
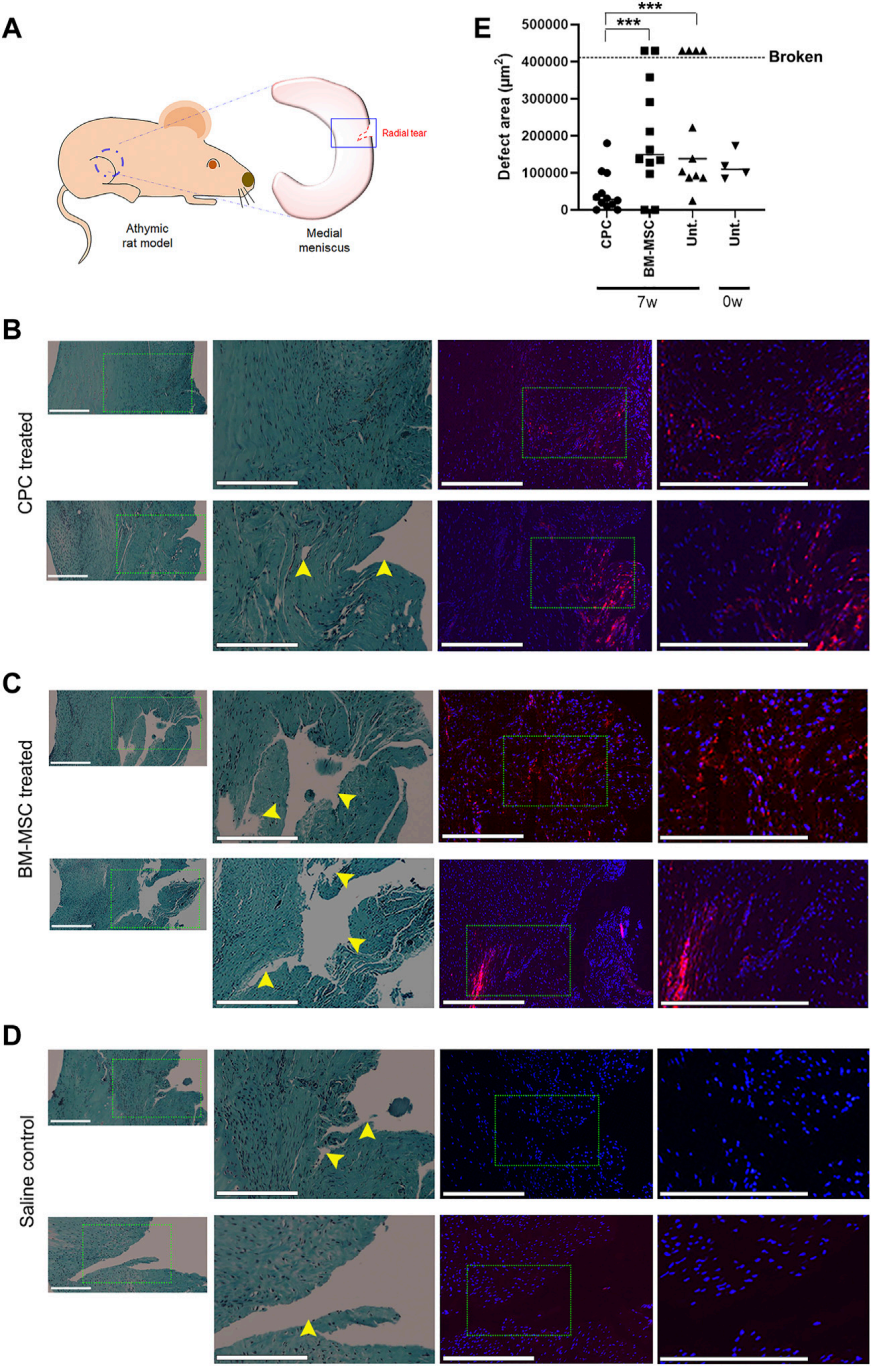
Intra-articular injection of CPCL3 following meniscus tearing stimulates fibrocartilage restoration and healing. **(A)** A radial tear was created in the outer third of the medial meniscus of skeletally mature athymic rats as shown in the diagram. Histology of meniscus tears 7 weeks following treatment with **(B)** CPCL3, **(C)** BM-MSCL, or **(D)** saline vehicle alone is shown. Left panels show sections stained with Safranin O/Fast green, while fluorescence imaging panels represent a sister section taken ∼150—300 µm away from the aforementioned section that has been stained with Dapi. Rightmost fluorescent panels are higher magnification images of the select area inscribed by the dotted line in the Dapi stained image. Nuclei of all cells (both native meniscus cells and injected cells) are stained with Dapi (blue). In the cell treatment groups, a total of 3.2 million fluorescently labeled cells (red) were injected into the joint capsule following meniscus injury. **(E)** Open areas remaining within the meniscal tear channel was quantified by image analysis and compared across experimental groups. A 0-week control group was used to determine the open area of the meniscal tear before healing, or further tearing due to the *in vivo* environment, can take place. Data points above the dotted line indicates menisci that were broken in two at the time of harvest. Representative images in panels B, C and D were not obtained from these broken menisci samples. Scale bars represent 400 µm. N ≥ 8 per group. ***, *p* ≤ 0.005.

To test our hypothesis that CPCs can accelerate meniscus tear repair in live animals, we implemented a medial meniscus tear model in RNU nude rats. A 1.0 mm radial tear was surgically created in the medial meniscus of the right hind limb, making sure that this injury did not sever the meniscus into two separate pieces (Figure 3A). Animals received an intra-articular injection of either CPCL3 (group 1), BM-MSCL (group 2), or saline alone (vehicle control, group 3). Cell treatments were divided into two separate injections (1.6 × 10^6^ cells each) administered at Week 1 and Week 4, post injury. All animals were euthanized 7 weeks post-injury and the medial menisci from the surgical knee was carefully excised for analysis. All meniscal samples were fixed in their neutral resting position on a flat surface to prevent contortion of the injury site prior to embedding for sectioning. Samples were then uniformly sectioned along the transverse plane to reach the widest region, which allowed assessment of the extent of injury with respect to the total width of the meniscus.

Histology sections were used to qualitatively visualize the injury/repair site (Figures 3B–D). Outcomes revealed that none of the animals in the CPCL3-treatment group had medial menisci that were broken into two separate pieces upon harvest, whereas 36.4% of rats in the vehicle control group, and 16.7% in the BM-MSCL treatment group were, suggesting that the injury worsened over time in these animals (Table 2). Moreover, we quantified the remaining open area of the meniscal tears, in each experimental group (Figure 3E). Four animals were euthanized immediately following meniscal injury (at 0 weeks), to measure the open area of the injury immediately after it was created. This was done for two reasons: 1) To quantify the initial size of the injury before it has had a chance to heal or become worse with knee usage; 2) To validate the reproducibility of the tear size from one surgery to the next. Outcomes showed that the extent of tissue reunification at the tear site was greatest in the CPCL3-treatment group, because it exhibited the least average open area. Since it is not possible to measure the exact open area in a completely severed meniscus, specimen that were found to have separated into two pieces were interpreted to have 20% larger open area than the largest unsevered meniscus that was observed in the study. We did this because it is a conservative adjustment that allows for the incorporation of these important outcomes into our quantitative analysis.

**TABLE 2:** Prevalence of broken medial menisci by treatment condition. The prevalence of broken medial menisci differed by treatment condition. The saline control group experienced significantly more broken defects than the CPCL3-treated group (36.4% vs 0%, *p* < 0.0001). There were no statistically significant differences in the prevalence of broken menisci in the saline control compared to BM-MSCL group (*p* = 0.30).

Saline	BM-MSCL	CPCL3	Comparisons			
			*p*-values	Saline vs. BM-MSCL	Saline vs. CPCL3	BM-MSCL vs. CPCL3
4 (36.4%)	2 (16.7%)	0 (0%)	Unadjusted	0.30	<0.0001	<0.0001
			Adjusted	0.30	<0.0001	<0.0001

### Posttraumatic cartilage degradation is significantly reduced following CPC treatment of meniscal tears

**FIGURE 4:**
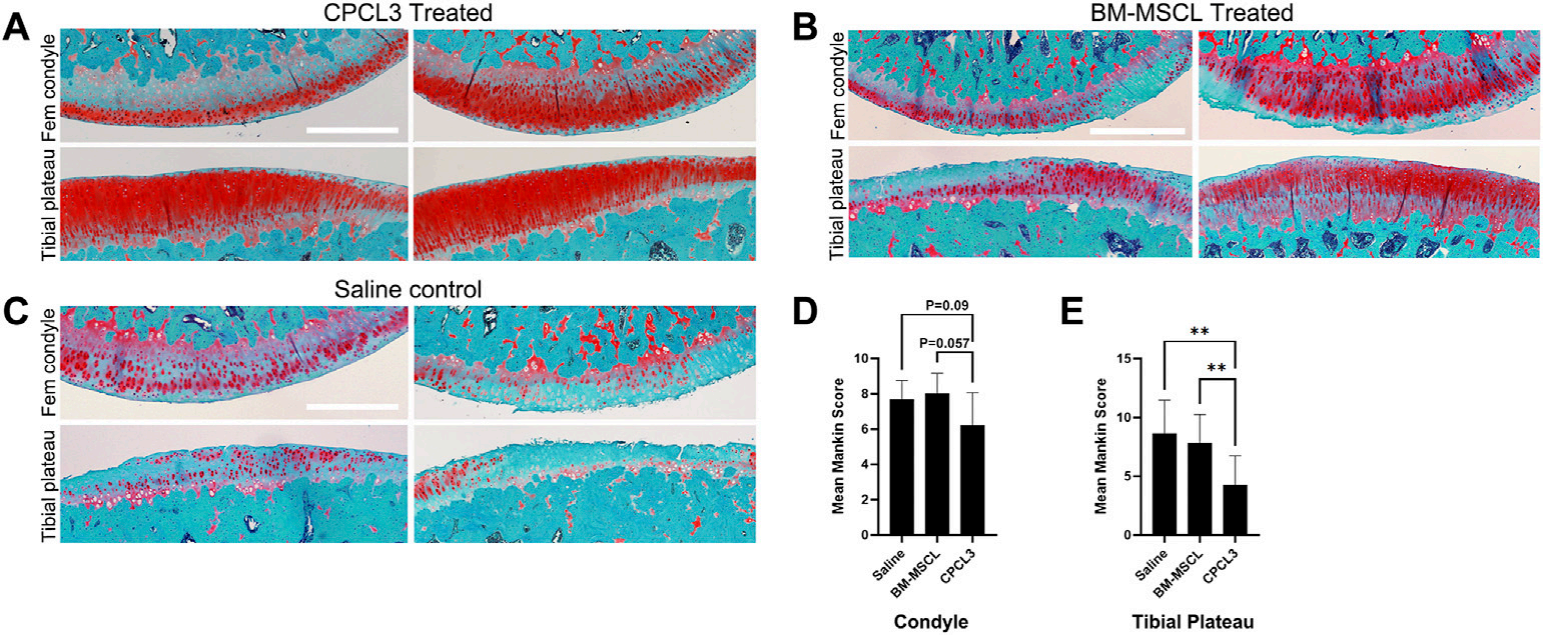
Animals with meniscus tears that were treated by intra-articular injection of CPCL3 exhibit reduced post-traumatic cartilage degeneration. The knee articular cartilage from the medial compartment of each knee that was subjected to a medial meniscus injury were sectioned and stained with Safranin O/Fast Green to visualize the extent of cartilage erosion. Staining of the medial femoral condyles and tibial plateaus of **(A)** CPCL3-treated, **(B)** BM-MSCL-treated, and **(C)** vehicle alone treated animals are shown. Both BM-MSCL-treated and vehicle alone treated animals show severe proteoglycan loss, indicated by diminished red staining in comparison to CPCL3-treated animals. Scale bars represent 500 µm. **(D)** Average modified Mankin scoring results of medial femoral condyles and **(E)** medial tibial plateaus of each experimental group are shown. N ≥ 8 per treatment group. **, *p* ≤ 0.01.

**FIGURE 5:**
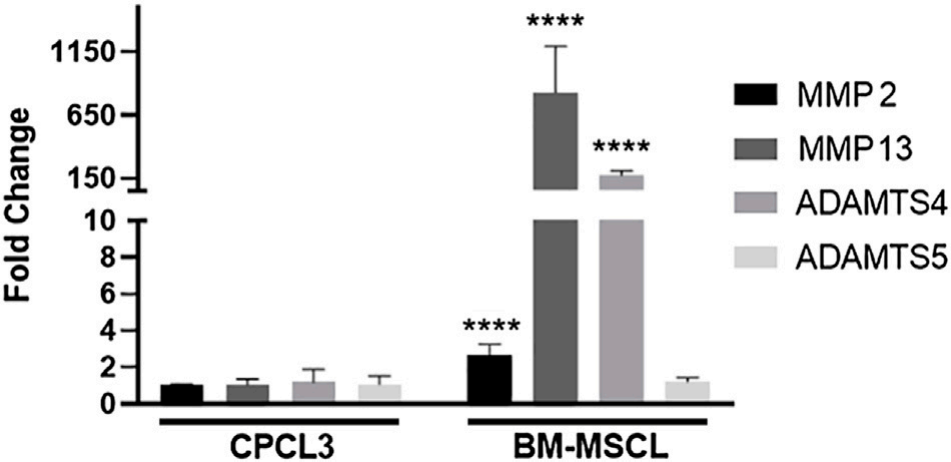
CPCL3 exhibits significant reduction in OA-associated catabolic enzyme expression, relative to BM-MSCL. Relative mRNA expression of collagenases and aggrecanases were compared between CPCL3 and BM-MSCL. Expression of *MMP2*, *MMP13*, and *ADAMTS4* are all elevated in BM-MSCL, while only *ADAMTS5* remained expressed at a comparable level between CPCL3 and BM-MSCL. N = 5 per group. ****, *p* ≤ 0.0001.

Articular surfaces of the injured and treated knees were inspected for signs of degradation resulting from the meniscal injury (Figures 4A–C). Independent blinded histopathology assessment was performed on the medial femoral condyles and medial tibial plateaus using a modified Mankin scoring system (Figures 4D,E). Mean femoral condyle scores showed that CPC-treated group exhibited improvement over the BM-MSCL-treated group and the saline control group. Tibial plateau scoring results indicated a significantly lower average Mankin score in the CPC-treated group compared to the vehicle alone control group, as well as the BM-MSCL-treated group, suggesting improvements in cartilage integrity in the CPC-treated animals. Relative basal mRNA expression of OA-associated proteinases *MMP2, MMP13, ADAMTS4,* and *ADAMTS5* was compared between CPCL3 and BM-MSCL (Figure 5). *MMP2, MMP13,* and *ADAMTS4* were significantly downregulated in CPCL3, suggesting that this reduction in catabolic enzyme production too may be a contributing factor to the difference in OA severity observed between these two treatment groups. Taken together with our previously published data demonstrating that *SOX9* is upregulated in CPC lines compared to BM-MSCs (Jayasuriya et al., 2019), this suggests that CPCs are more anabolic and less catabolic than BM-MSCs, which may contribute to their improved efficacy for stimulating meniscus repair and preventing cartilage degradation.

## Discussion

Cell-based meniscus repair strategies are currently being investigated using several musculoskeletal tissue specific mesenchymal cell sources; including progenitors derived from meniscus tissue itself, as well as synovium, adipose, and cartilage (Muhammad et al., 2014; Seol et al., 2017; Sun et al., 2020; Twomey-Kozak and Jayasuriya, 2020; Trivedi et al., 2022). CPCs are a promising cell-type for musculoskeletal tissue engineering applications given their inherently high chondrogenicity, propensity for colony formation, and resistance to chondrocyte hypertrophy (Dowthwaite et al., 2004; Fickert et al., 2004; Williams et al., 2010; McCarthy et al., 2012; Vinod et al., 2020). Further, these cells can be extracted from an available cell source (non-loadbearing cartilage), which is currently what is used for autologous chondrocyte implantation (ACI) (Ossendorf et al., 2011). However, a logistical hurdle for implementing these cells for therapy is their low natural abundance and the need to obtain sufficient expendable healthy cartilage tissue from which they can be extracted. Further, what little primary CPCs can be extracted from cartilage must undergo *in-vitro* expansion to obtain sufficient cell numbers for therapeutic use, which runs the risk of diminishing their proliferative capacity due to gradual replicative senescence that begins to set in during the extensive multiple-passage expansion of mesenchymal progenitor populations (Banfi et al., 2000; Baxter et al., 2004). These hurdles exist whether one seeks to use autologous (from same patient) or allogeneic (donor-derived) cell sources, unless a large amount of tissue can be made available from either source, which is rare. Here, we utilize a previously established stable CPC cell line (Jayasuriya et al., 2019; Newberry et al., 2020; Twomey-Kozak et al., 2020) to perform a proof-of-concept study set to test their therapeutic efficacy for fibrocartilage injury repair in a live animal model. The goal of this study was to determine whether a stabilized clonal CPC line can be used as an available alternative cell source for musculoskeletal joint tissue repair.

Prior to administering them into animals, the cell surface marker profiles and proliferative capacity of CPCL3 and the BM-MSCL control cell line, were examined. No significant differences in surface markers were found in both cell lines confirming that this aspect of their cell phenotype was consistent with their primary counterparts. When we compared the innate proliferation rate of the stable cell lines and primary cells, we discovered that even lightly expanded primary progenitor cells (passaged 4–6 times) exhibited significantly diminished proliferative capacity relative to the stable lines, both of which had been expanded from 7–10 passages, after the stabilization procedure. We also observed that CPCs consistently showed increased proliferative capacity, compared to BM-MSCs in the same class (i.e., stable CPC line compared to stable BM-MSC line; primary CPCs compared to primary BM-MSCs).

While our overall findings suggested a logistical advantage in using stable cell lines for therapy, due to their improved proliferative capacity, we carefully inspected whether these cells would proliferate uncontrollably in the joint space and/or migrate outside the joint capsule and engraft in off-target locations, such as vital organs in our athymic rat model. Gross inspection of the surgical knees of animals treated with either CPCL3 or BM-MSCL at the experimental endpoint of 7 weeks following surgery, showed the absence of any abnormal growths. Similarly, gross inspection of vital organs (kidney, liver, spleen, lungs, and brain) at the time of joint harvest revealed no such irregularities. As a secondary confirmation, DNA collected from homogenized organs were used as templates to run PCR using primers that only recognize and bind human DNA (specifically, the human Cytochrome B gene as done by Matsuda et al. (2005)). Results indicated no detection of this ubiquitous human-specific gene, signifying the absence of human cells (i.e., CPCL3/BM-MSCL) in these tissues (Supplementary Figure S3).

Intra-articular injection of stable CPCs significantly improved both meniscal tear healing and cartilage health outcomes in animals, compared to BM-MSCL treated animals. This strongly suggests that CPC treatment can safeguard against PTOA changes in the knee, following meniscal injury. We were surprised to find that in all performed outcome measures, the BM-MSCL treated animals showed no significant improvements over the control animals that were given vehicle only. To better understand why there was such a significant difference in the treatment efficacy between CPCL3-treated and BM-MSCL-treated animals, we analyzed the expression profile of collagenases and aggrecanases that are produced by each cell type. We focused specifically on these catabolic genes because they are involved in cartilage turnover, and their differential expression may help to explain why CPCL3-treated animals showed less severe cartilage erosion. Our findings showed that CPCL3 exhibited significantly lower expression of catabolic proteinases *MMP2, MMP13,* and *ADAMTS4* compared to BM-MSCL. Relative to CPCL3, BM-MSCL exhibited >800-fold expression of *MMP13*, which has been reported to be the most potent cartilage eroding proteinase (Mitchell et al., 1996). Similarly, BM-MSCL exhibited 175-fold expression increase in *ADAMTS4*, which is also a prominent catabolic enzyme responsible for cartilage breakdown (Westling et al., 2002).

Interestingly, fluorescence imaging of meniscal sections from both cell treatment groups showed a wider distribution of administered cells throughout the meniscus than we expected. For instance, in some cases, cells were not found directly on the banks of the remnant tear channel, but rather they were found adhering to nearby adjacent regions of meniscus tissue. In the case of CPCs, this observation suggests that the mechanism by which these cells promote meniscus healing may (at least in part) involve their exertion of paracrine effect(s) that inspire native meniscal cells to form neo tissue and reunify the torn region. Identifying and examining these detailed mechanisms will be the subject of future investigation.

The athymic RNU rat model was selected for this proof-of-concept study to minimize any potential immune reaction/rejection due to the administered human cells. While we acknowledge that this is a study limitation, we opted for this model because it enabled us to first determine whether the stable cell therapies are capable of stimulating meniscal healing in an *in vivo* knee microenvironment, without having to consider immuno-rejection as an added complication. In the future, we anticipate designing studies that will be conducted in fully immunocompetent and large animals (both males and females), to further test the efficacy and translatability of this approach. Although we demonstrated that stable CPCs have higher proliferative capacity over primary CPCs, mRNA analysis revealed that CPCL3 exhibited detectably lower collagen I and II expression compared to primary CPCs, despite having higher *SOX9* expression. This may be considered a potential disadvantage of utilizing the stable CPC line for musculoskeletal tissue repair, over using primary CPCs. Further, future studies should focus on evaluating the long-term efficacy of using immortalized cells for cell-based therapy. Another limitation of our study was our inability to perform histological evaluation of the joint synovium, since the joint could not be kept intact considering that we needed to carefully excise the medial meniscus in order to conduct our quantitative analysis of the tear site, which required sectioning the tissue in the transverse plane. This means that the joint needed to be disarticulated, which ruptured the synovial capsule during sample preparation.

In conclusion, this is a proof-of-concept study demonstrating that a stable human cartilage progenitor cell line can facilitate meniscus tear healing and significantly reduce the severity of PTOA changes in knee articular cartilage. Futures studies will focus on evaluating the long-term safety and efficacy of utilizing stable CPCs for meniscus tissue repair. We will also determine safety and efficacy using an immunocompetent model. Lastly, we will explore strategies to scale up the success that we report here in rodents to a clinically relevant large animal model, similar in size to human patients. If success is demonstrated in the above anticipated studies, the opportunities for advancing the current state of cell-based biologic therapy for musculoskeletal tissue repair stands to gain significant ground. The implication of having a readily available stable cell source, that does not require an initial surgery to secure, that can be cryopreserved and kept for safe and effective repair for treating traumatic meniscus tears would dramatically change the way these injuries are currently addressed in the clinic.

## Data Availability

The raw data supporting the conclusions of this article will be made available by the authors, without undue reservation.

## Supplementary material

Supplementary Figure S1 Side-by-side comparison of the cell surface marker profiles of the stable BM-MSC line (BM-MSCL) and primary BM-MSCs. Cell surface marker expression rates of **(A)** Fibronectin, **(B)** Thy-1, **(C)** Endoglin, **(D)** ALCAM, and **(E)** ICAM1 in BM-MSCL and primary BM-MSCs. Primary BM-MSCs were obtained from American Type Culture Collection, which dispenses cells collected from healthy human tissues. The empty peaks represent isotypic controls, for reference. Percent values indicated in the graphs represent positive staining rate, above isotype control, for each individual CD marker.

Supplementary Figure S2 Comparison of chondrogenic marker expression and cell proliferation capacities of BM-MSCL and primary BM-MSCs**.** Fold differences in the mRNA expression of **(A)** *SOX9*, **(B)** *COL2A1*, and **(C)** *COL1A1* between primary BM-MSCs and the stable BM-MSC line, BM-MSCL. N≥4 per group. ****, P≤0.0001. **(D)** Cell proliferation assay results showing the proportion of proliferating cells in BM-MSCL cultures and primary BM-MSC cultures.

Supplementary Figure S3 Off-target engraftment of stable human cell lines was not detected in the major organs of cell-treated athymic rats. Agarose gel electrophoresis was used to confirm that human DNA was absent in major organs that are common targets for cell engraftment and metastasis. Tissues from cell-treated animals were homogenized and the total DNA was used as templates for PCR amplification of the human mitochondrial cytochrome C gene. DNA that was directly extracted from each line was subjected to the same procedure described above and used as internal positive controls. Observed bands are marked with arrows. DNA ladder is labeled with “L”. Data is representative of results observed in all experimental animals in each respective cell-treatment group.
