# Supplementary figures and images for: Stable human cartilage progenitor cell line stimulates healing of meniscal tears and attenuates post-traumatic osteoarthritis
Source: Front Bioeng Biotechnol. 2022 Oct 12;10:970235. doi: 10.3389/fbioe.2022.970235 (PMC9596807; doi:10.3389/fbioe.2022.970235)

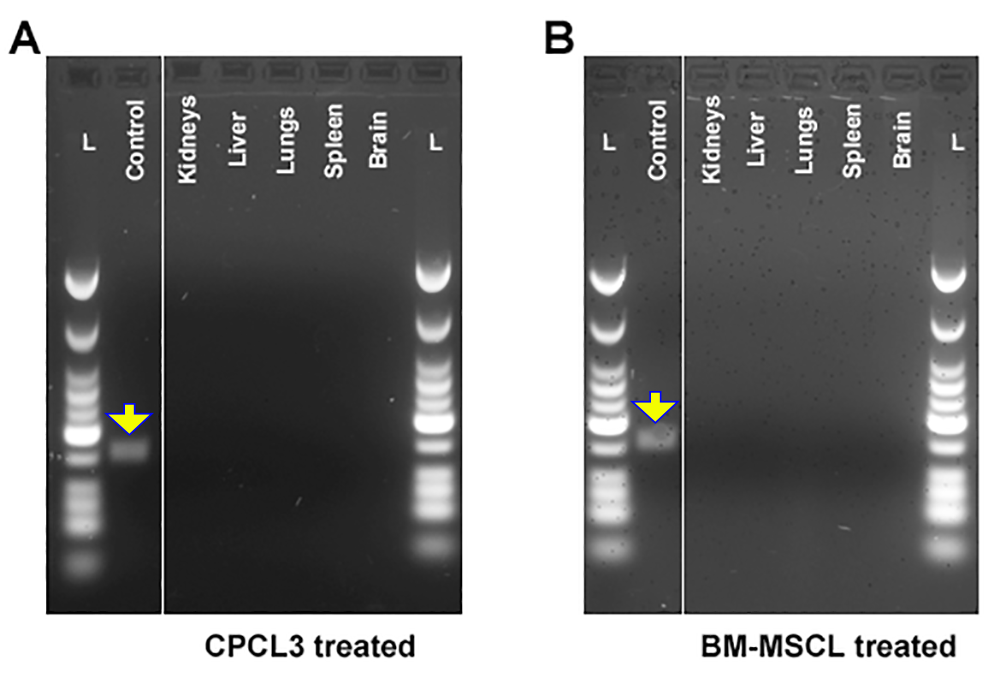

Supplement: Supplementary file 1 [file Image3.TIF]

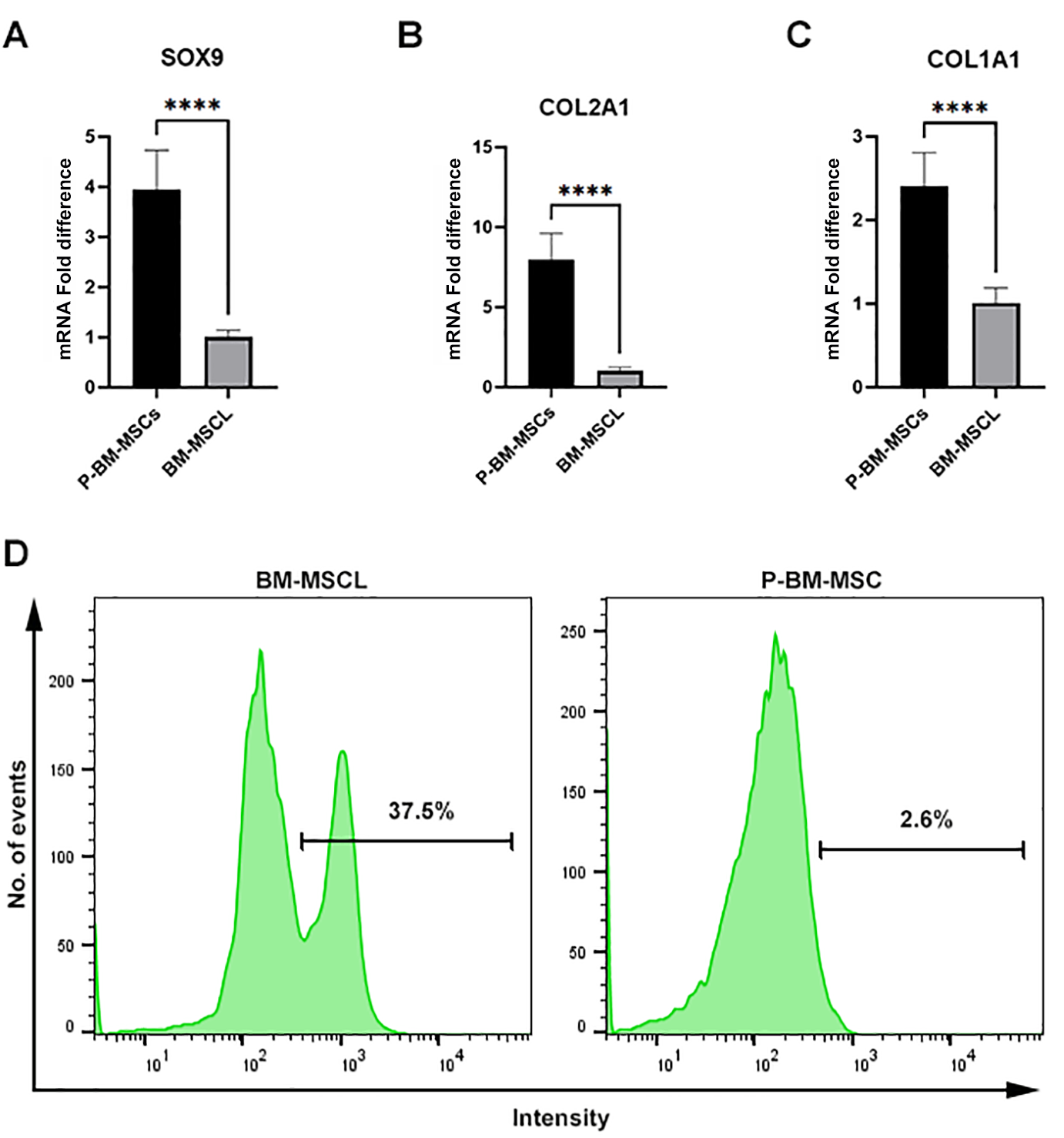

Supplement: Supplementary file 2 [file Image2.TIF]

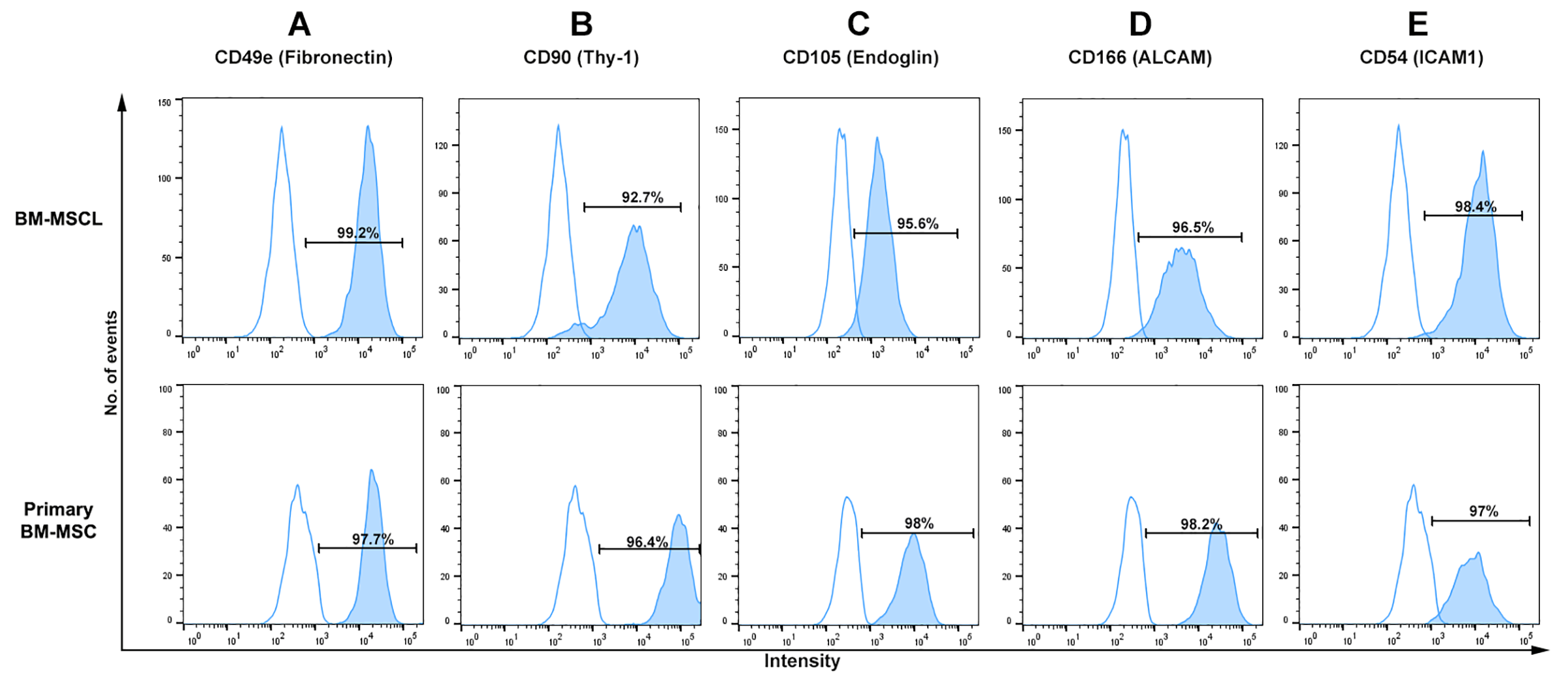

Supplement: Supplementary file 3 [file Image1.TIF]
